# Supplementary material for: A Pan-Cancer Analysis of Cystatin E/M Reveals Its Dual Functional Effects and Positive Regulation of Epithelial Cell in Human Tumors
Source: Front Genet. 2021 Sep 17;12:733211. doi: 10.3389/fgene.2021.733211 (PMC8484784; doi:10.3389/fgene.2021.733211)
Supplement: Supplementary file 1 [file Data_Sheet_1.PDF]

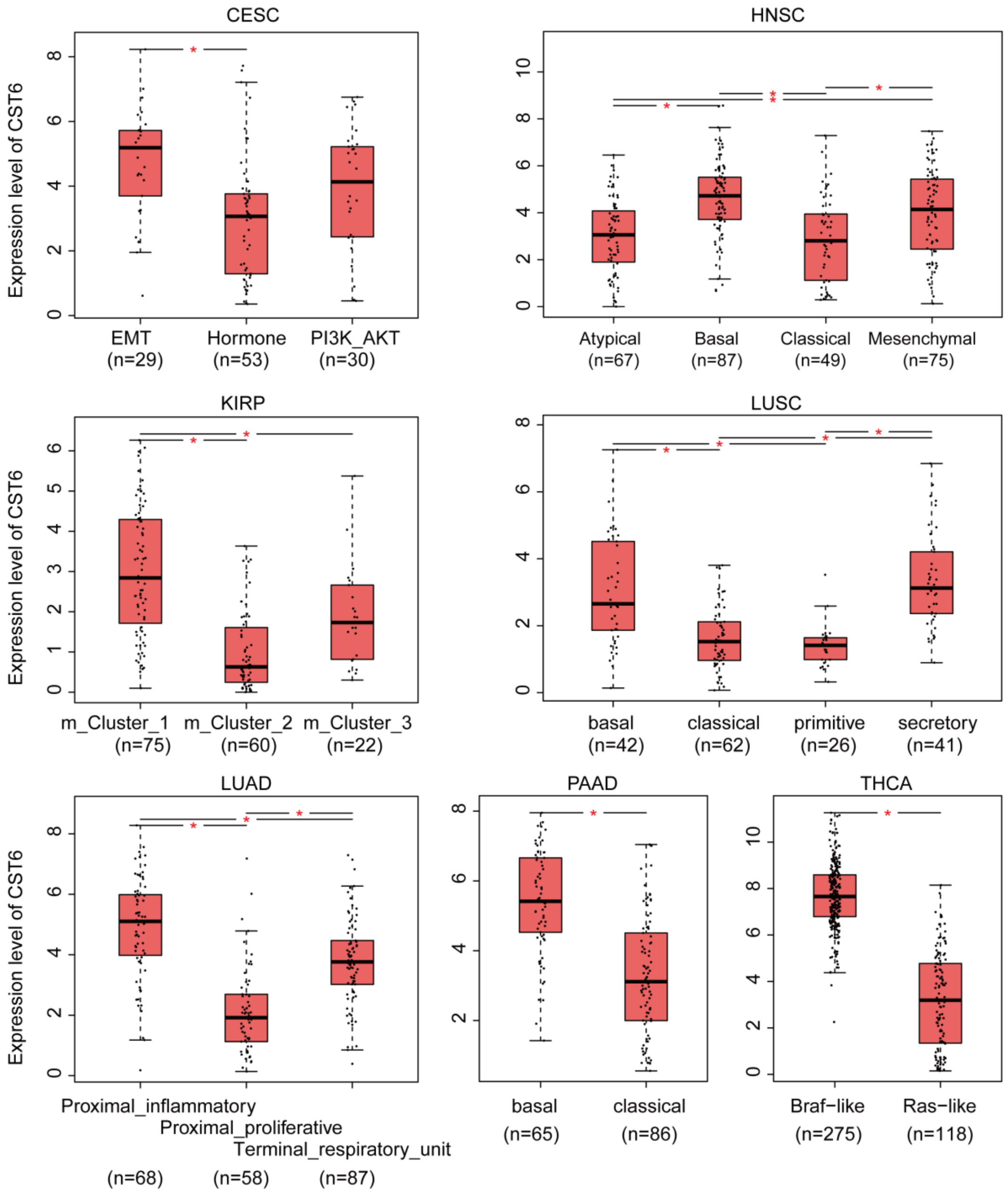

Figure S1. Expression level of CST6 was related to the subtype of CESC, HNSC, KIRP, LUSC, LUAD, PAAD and THCA cancer types.

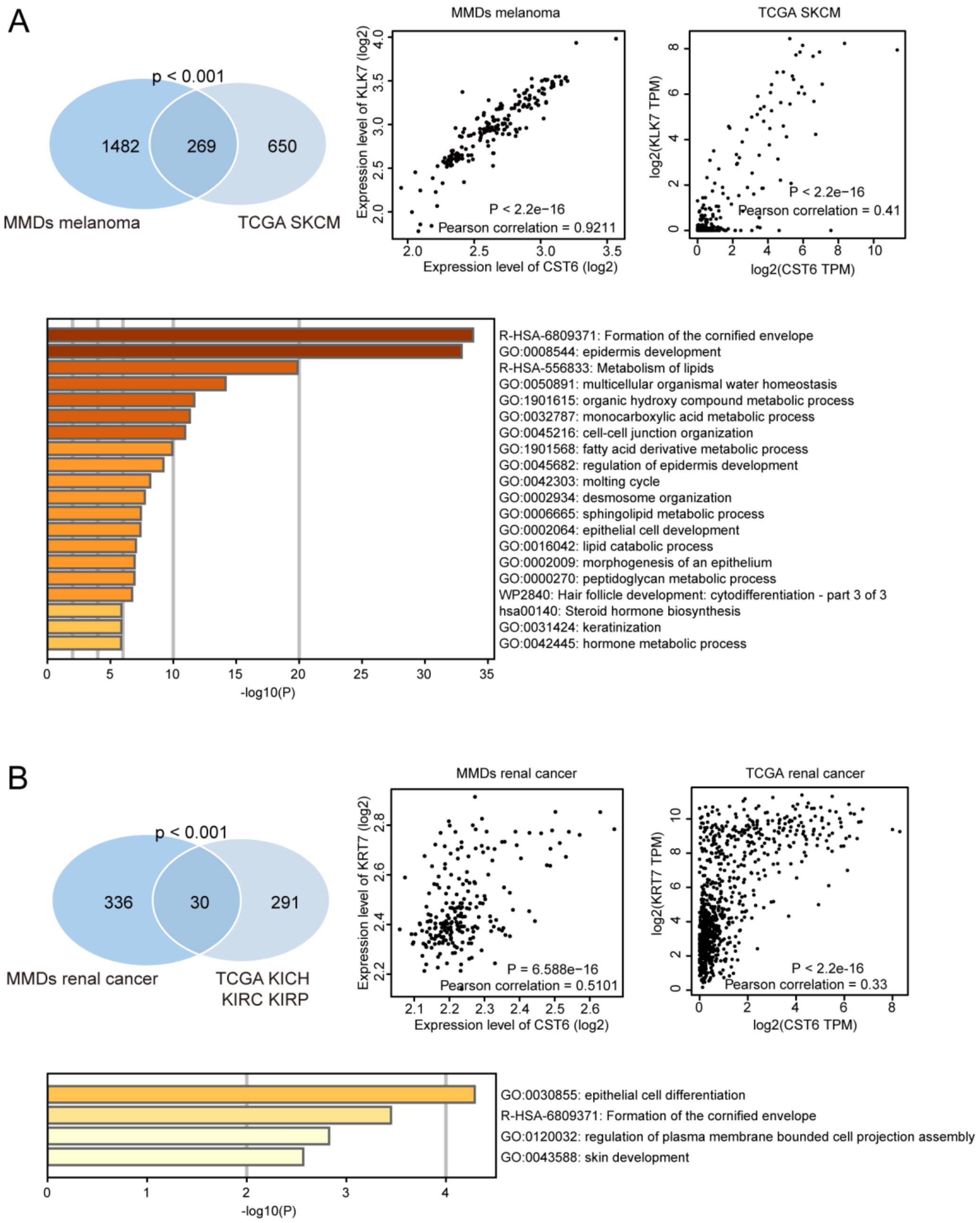

Fig S2. Identification and functional analysis of CST6-related genes in melanoma and renal cancer datasets. (A) Overlap of CST6 genes and functional analysis between MMDs melanoma and TCGA SKCM datases. (B) Overlap of CST6 genes and functional analysis between MMDs renal cancer and TCGA renal cancer (KICH, KIRC and KIRP) datases.

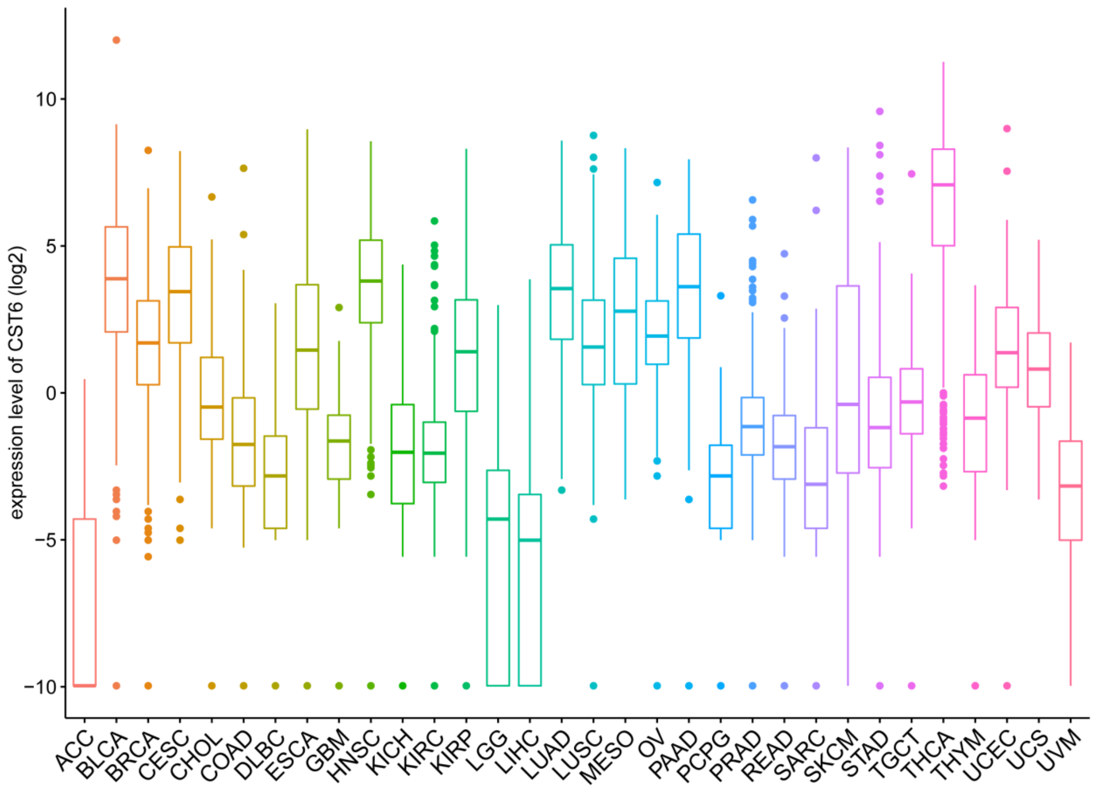

Figure S3. The expression level of CST6 across TCGA cancer types.

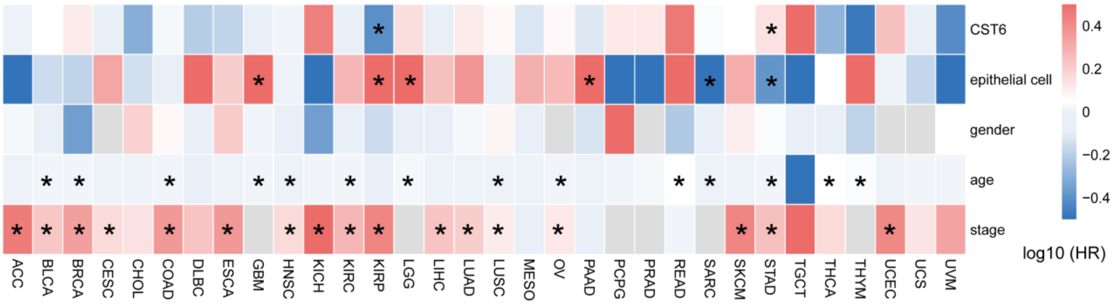

Fig S4. Multivariate survival analysis considering CST6 expression, epithelial cell score, gender, age and stage in TCGA datasets. * represents p value < 0.05. Rectangle colored gray represents NA.

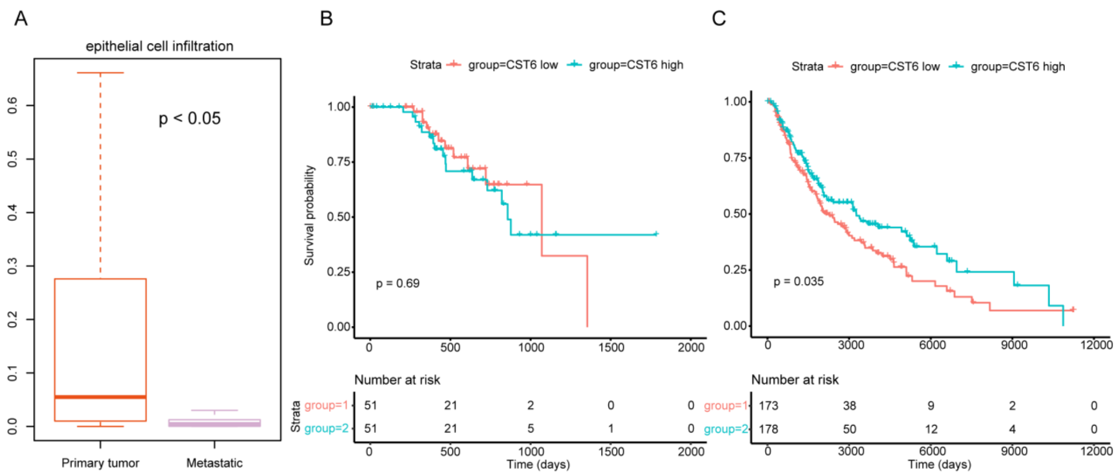

Fig S5. Comparison of epithelial cell infiltration and clinical survival between TCGA SKCM primary tumor and metastatic patients. (A) Comparison of epithelial cell infiltration between TCGA SKCM primary tumor and metastatic patients. (B) Kaplan-Meier estimates of overall survival by CST6 expression in TCGA SKCM primary tumor patients. (C) Kaplan-Meier estimates of overall survival by CST6 expression in TCGA SKCM metastatic patients.

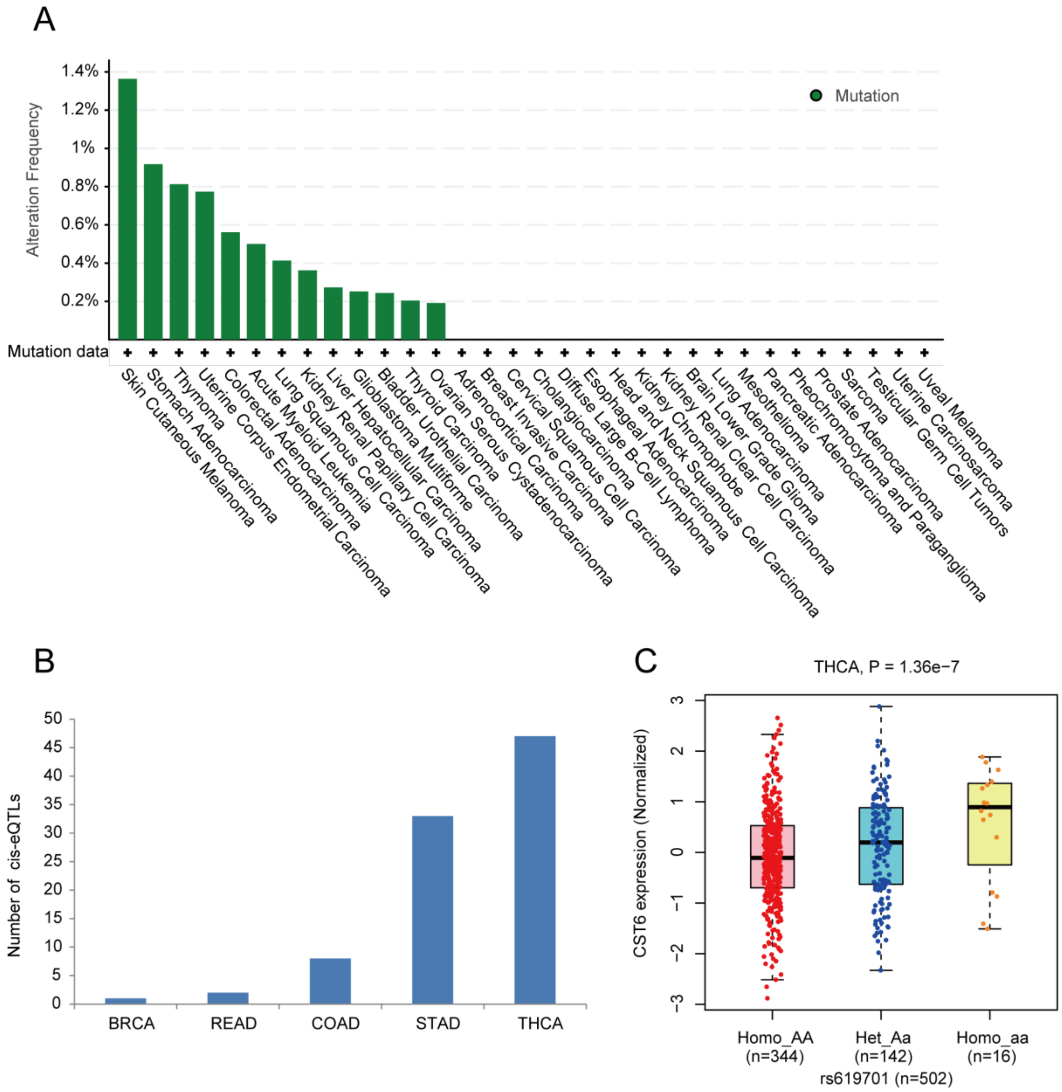

Figure S6. Mutation feature of CST6 across TCGA cancer types. (A) The mutation frequency of CST6 across TCGA cancer types. (B) The number of CST6-related cis-eQTL across TCGA cancer types. (C) The influence of rs619701 alternation on the CST6 expression for THCA patients.

Table S1. Number of samples in MMDs datasets

cancer type	cancer number	normal number
bladder	161	21
breast	2088	214
colorectal	1393	121
gastric	691	46
liver	264	137
lung	1474	147
melanoma	194	20
ovarian	593	54
pancreatic	108	70
prostate	121	116
renal	219	104

Table S2. Detailed information of TCGA cancer types

cancer type	full name
ACC	adrenocortical cancer
BLCA	bladder urothelial carcinoma
BRCA	breast invasive carcinoma
CESC	cervical and endocervical cancer
CHOL	cholangiocarcinoma
COAD	colon adenocarcinoma
DLBC	diffuse large B-cell lymphoma
ESCA	esophageal carcinoma
GBM	glioblastoma multiforme
HNSC	head and neck squamous cell carcinoma
KICH	kidney chromophobe
KIRC	kidney clear cell carcinoma
KIRP	kidney papillary cell carcinoma
LGG	brain lower grade glioma
LIHC	liver hepatocellular carcinoma
LUAD	lung adenocarcinoma
LUSC	lung squamous cell carcinoma
MESO	mesothelioma
OV	ovarian serous cystadenocarcinoma
PAAD	pancreatic adenocarcinoma
PCPG	pheochromocytoma and paraganglioma
PRAD	prostate adenocarcinoma
READ	rectum adenocarcinoma
SARC	sarcoma
SKCM	skin cutaneous melanoma
STAD	stomach adenocarcinoma
TGCT	testicular germ cell tumor
THCA	thyroid carcinoma
THYM	thymoma
UCEC	uterine corpus endometrioid carcinoma
UCS	uterine carcinosarcoma
UVM	uveal melanoma

Table S3. Number of samples in expression and methylation datasets from TCGA database

cancer	Exp (C)	Exp (N)	Methy (C)	Methy (N)	Common (C)	Common (N)
ACC	77	NA	79	NA	76	NA
BLCA	407	19	412	21	407	17
BRCA	1092	113	779	87	775	74
CESC	304	3	306	3	303	3
CHOL	36	9	36	9	36	9
COAD	288	41	288	34	271	17
DLBC	47	NA	48	NA	47	NA
ESCA	181	13	183	15	179	10
GBM	153	5	139	2	51	1
HNSC	518	44	523	50	513	20
KICH	66	25	65	NA	65	NA
KIRC	530	72	312	157	309	23
KIRP	288	32	271	43	268	23
LGG	509	NA	514	NA	507	NA
LIHC	369	50	374	50	366	41
LUAD	513	59	456	30	451	20
LUSC	498	50	364	41	362	8
MESO	87	NA	87	NA	87	NA
OV	419	NA	10	NA	9	NA
PAAD	178	4	183	10	177	4
PCPG	177	3	178	3	176	3
PRAD	495	52	495	50	492	35
READ	92	10	94	7	87	2
SARC	258	2	257	4	254	NA
SKCM	102	1	104	2	102	1
STAD	414	36	393	2	369	NA
TGCT	148	NA	149	NA	147	NA
THCA	504	59	503	56	500	50
THYM	119	2	124	2	119	2
UCEC	180	23	418	45	173	23
UCS	57	NA	57	NA	57	NA
UVM	79	NA	80	NA	79	NA

Exp (C): number of cancer samples for expression dataset; Exp (N): number of normal samples for expression dataset; Methy (C): number of cancer samples for methylation dataset; Methy (N): number of normal samples for methylation dataset; Common (C): number of common samples for expression and methylation in cancer datasets; Common (N): number of common samples for expression and methylation in normal datasets.

Table S4. Detailed information of CST6-related genes.

Cancer type	Gene list
Lung cancer	ITGA3, ATP10A, CATSPER1, CMTM3, TNFRSF12A, PTTG1IP, AREG, KCNN4, EHD1, SFXN3, SAMD4A, ARPC1B, SNX21, VASN, SDC4, AMIGO2, FHOD1, PHLDA2, ZNF341, VDR, SIPA1, ARL6IP5, BEAN1, MDFIC, CRIP1, RGS3, SRPX2, RGS10, CLCF1, ACTB, PON2, UNC13D, MFSD7, CDA, CCM2, AGRN, BATF3, DYSF, TSPAN15, S100A10, CRIP2, MAP7D1, MROH6, CTSA, IFNAR2
Melanoma	KLK7, WFDC5, DSG1, SDR16C5, RAB25, DSC3, CLCA2, SBSN, EPHX3, BNIPL, TRIM29, ARG1, CALML3, DSC1, GRHL1, TMEM40, C1orf116, PKP1, S100A14, BBOX1, ZNF750, SULT2B1, POF1B, CYP4F22, HAL, GPR87, BPIFC, ESRP2, SCEL, ENDOU, ABCA12, EHF, CWH43, TMEM79, SPINK5, MUCL1, GGT6, DMKN, EPN3, PRSS8, HOPX, IL22RA1, GJB3, KRT80, OVOL1, SLC46A2, DUOXA1, KRT5, TACSTD2, TMEM45B, GRHL3, ADGRF4, IL20RA, GJB5, CDS1, JUP, SCNN1B, CHP2, LAD1, PKP3, SH3RF2, ACER1, TP53AIP1, CHMP4C, UNC93A, MAL2, MPP7, SDR9C7, KRT10, PROM2, RAPGEFL1, SMPD3, MPZL3, EVPL, FAAH2, ELOVL4, POU2F3, DGAT2, RORA, A2ML1, PTK6, IRF6, KCNK7, GATA3, DUOX1, TNS4, C1orf106, KLC3, EPHB6, CASP14, SPINT2, SLC15A1, SCNN1A, ATP6V1C2, CIDEA, SLC5A1, PPL, LYPD6B, ALDH3A1, CD207, CERS3, SLC39A2, PPP1R13L, SOX7, TTC39B, FAM83A, HSD17B2, KRT79, KDF1, EPHA1, AP1M2, SGPP2, XG, GRHL2, TP63, DLX3, EXPH5, RHBDL2, MAPK13, RHOV, ARHGEF4, CLTB, SDR42E1, ST14, UGT1A6, SYTL1, RAB27B, KRT31, NLRX1, PYDC1, DSP, TMEM154, ELMO3, IDE, CTSV, CD1A, TMPRSS11E, GPT2, ATP12A, ABHD5, STAP2, MARVELD2, KRT34, DNASE1L2, OTUB2, SLC1A6, ALOX12, MICALCL, MAP7, C2orf54, SDCBP2, TGM5, RAET1E, CPA4, S1PR5, IMPA2, SPRR4, KRT75, SLC26A9, BCL2L10, PNPLA1, GDA, ADTRP, GLTP, NKPD1, TM7SF2, CRB3, TMEM45A, GJA1, TMEM254, PIK3C2G, ZC3H12A, EPB41L4B, LLGL2, KLF4, CASZ1, VSNL1, CHI3L2, SERPINB12, PERP, FA2H, ELOVL3, CYP4F3, CTNNBIP1, SP6, TMEM184A, SUSD4, FRMPD1, CYB5A, SRD5A1, TMEM91, CLDN8, PRRG2, GRB7, PADI1, CXCR2, ALOX15B, DHCR24, ME1, PLEKHG6, DAPK2, FETUB, IL1RL2, KRT26, CLDN4, PLA2R1, C11orf52, AGR2, MAB21L3, ATG9B, KLB, SPTLC3, AACS, MSMO1, ARSF, THRSP, SLCO4C1, PGLYRP4, CYP3A5, KRT71, CYP4F2, PXMP4, TST, KRT32, KRT27, HMGCS1, GPT, RDH16, KRT74, MVD, KRT85, PM20D1, ADGRL3, CYP4F12, PGLYRP2, BARX2, FOXN1, LYG2, CCDC120, GAL, GDPD2, LIPH, CYP4F8, ACAD8, KRT72, PGLYRP3, KRT36, ACSL1, SGK2, HSD11B1, PLA2G2F, KRT73, FAM26E, TTC22, LDHD, CRAT, HAO2, TJP3, PDZK1, MARVELD3, BPY2C, S100A3, DCD, UGT2A1, KRTAP17-1, VTCN1, BRI3BP, PLA2G4D, AR
Renal cancer	KRT7, EPS8L1, TACSTD2, SCEL, LAMA3, GJB3, TJP3, SH2D3A, SYNE4, MST1R, IQCD, CCNO, ANXA3, ELF3, PRRG2, LRRN4, MAL, CLDN7, B3GNT7, CRCT1, PPL, C11orf49, KRT6B, MAL2, HGFAC, HSD11B1L,

	ZMYND10, ST6GALNAC5, KCNS1, KIF9
Pan-cancer	DOCK9-AS2, NPC2, DCSTAMP, DAPK2, LCN12, RP11-474O21.5, GS1-114I9.1, TCERG1L, S100A5, PNPLA5, CRYGN, C2orf40, RP11-280O1.2, RP3-449M8.9, ARNTL, DHRS3, DTX4, GABRB2, NOD1, MIR181A2HG, SLC27A6, IGSF1, DNASE1L2, ZCCHC12, ATP13A4, APLP2, LPAR5, PDE5A, S100A13, TSHR, RP11-93H12.4, MGAT4C, WDR86, DOCK9, PDLIM4, RP11-44N11.2, ZBED2, SDC4, FN1, SLC17A5, RP11-20J15.3, SLC25A47P1, SNX22, RXRG, AC008940.1, RAPGEF3, EPHA4, NKX2-1, ZCCHC16, PPP1R14C, CTNNAL1, AF131215.8, CATSPER1, CLIC3, CTSB, ITGA9, PROS1, ARMCX3, NKX2-1-AS1, RP4-555L14.4, RPARP-AS1, RP11-216L13.16, DUSP5, SFTA3, SNX1, MUC15, DUOXA1, FSCN2, LONRF2, SHE, RP3-449M8.6, CITED1, NAB2, TMEM243, ST3GAL5, WDR86-AS1, AC007255.8, SHROOM4, AFAP1L2, ENTPD1, LMO7-AS1, CTC-441N14.2, RP11-542B15.1, RP11-44N11.3, AC090505.5, KCNJ2-AS1, ISCA2, FAM20A, DUSP6, LMO3, RP11-285E23.2, RMST, LCN10, SLC30A2, PRR15, IQCA1, S100A6, SYT12, INHBB, KISS1, ST3GAL5-AS1, TEPP, RP5-1065P14.2, TRPC5, LCN6, PDLIM1, C12orf49, LRP5L, RP11-302F12.1, SLC34A2, RP11-1036E20.9, AP000997.3, MFSD6L, RP11-789C17.5, MPZL2, KIAA1217, MAPKAPK3, ITGA3, ARMCX6, DGCR6, PTCSC3, PTPRM, TNFRSF12A, FRMD3, GGT2, RMST_10, VSTM4, PRMT8, DRAP1, RP11-548P2.2, SH3BGRL2, LINC00891, RMST_9, RP11-896J10.3, KCNJ15, PTCHD4, AK1, LCA5, ARMCX2, PPAP2B, NPNT, TCERG1L-AS1, PTCSC2, IGFBP6, PDE1A, ECE1, RP4-545K15.5, RGL3, WTIP, INMT, RP11-231P20.2, RP11-541H12.1, TRMT44, GGTLC3, KB-1183D5.13, PALM3, CTD-3076O17.1, RP11-13P5.2, RAP1AP, RP11-164P12.5, PRDM1, TNRC6C-AS1, RP11-532F12.5, AC002066.1, GGT3P, SIGLEC6, MRPS6, TBC1D2, TCTN3, COL8A1, KRT18P13, CTD-2182N23.1, CAV2, HCG22, SNX25, MXRA8, RP11-631N16.2, ZNF486, C10orf142, RP11-677M14.2, RP11-894P9.2, RMST_6, DUOX1, HIRA, ACVR1, IL1RL2, LTBP3, KCNJ16, AC008132.15, FUCA1, HHEX, AC144831.3, CYB561D2, RP11-768F21.1, RP1-134E15.3, SMIM1, LCN8, SNX5, VEGFC, KRT7, AF131215.6, TSTD1, OR4D6, GJA4, RP11-359G22.2, GSN, ERICD, RASA1, CTD-2619J13.13, RAB34, FANK1, CC2D2B, RP11-105N14.1, PRICKLE1, FOXE1, MIEF2, RP11-221N13.3, CPVL, TSC22D1, SMIM5, RP5-875H18.9, CLIP4, GGTLC5P, SLC5A3, CTF1, RASAL1, RP11-690P14.4, WI2-2610K16.2, PPP1R21, MPZL3, DOCK3, TCEB1P20, IL1RL1, KCNJ2, RP11-111E14.1, ADK, LINC01315, RP11-376P6.3, PAX8, RP11-547C5.1, UACA, RSPO4, FAM183DP, MBIP, MIR222HG, SSXP3, XXbac-B33L19.12, RP11-631N16.4, TPD52L1, GRAMD3, COL8A2, PLS3, GPR108, RILP, RP11-290H9.2, RP11-463I20.1, TNFRSF10C, LINC01483, EPOR, RP11-195F19.5, NPM2, NELL2, ANXA1, TMCC3, GAL3ST3, RP4-568C11.4, KRTAP9-3, KRTAP4-4, KRTAP10-7, KRTAP4-5, KRTAP12-2, KRTAP9-9, KRTAP10-5, KRTAP10-10, PLK3, KRTAP10-8, KRTAP9-7, KRTAP9-8, KRTAP10-3, KRTAP9-2, TMEM98,

	AWAT2, KRTAP10-1, KRTAP12-3, KRTAP4-7, RP11-166D19.1, KRTAP4-9, KRTAP9-11P, PLXNB2, KRTAP4-6, KRTAP10-9, KRTAP4-12, KRTAP4-3, FAM170B-AS1, KRT25, RRAS, KRTAP4-8, KRTAP10-11, KRTAP4-2, C15orf56, AADACL3, KRTAP12-1, BNIP3P15, RP11-302F12.2, AP001258.4, KRTAP10-12, KRTAP4-11, RECQL5, AWAT1, SLC38A5, ZNF430, SGMS2, SCEL, RP11-800A3.2, C2orf81, SAT1, KRTAP10-2, VPS13D, GTF2IRD1, KRTAP5-11, CHST2, SPACA6P, FAM3C, KRTAP9-4, MAP3K1, ALS2CL, C10orf55, KRTAP16-1, IFNWP19, HNRNPA1P27, KRTAP10-4, TDRP, KRT82, KRTAP2-1, SERGEF, KRTAP1-3, AC006539.2, PLA2G2E, MET, ZFAND2B, AC006022.4, HBEGF, C5orf67, RSPH6A, CPAMD8, CCDC148, AC005592.3, SYF2, PSG8, INPP5K, ITGB3, RP11-357P18.2, KRTAP24-1, RP11-91J19.3, LMBRD1, FABP9, BHLHB9P1, CTD-3128G10.6, BDH2, TENM1, KRTAP2-4, LTBP2, CDC42BPG, LRP4, VASN, NKX2-8, AC022431.3, BEAN1, RAB27A, TRIB1, CTC-236F12.4, CHD4, VIM-AS1, PDGFRL, NID2, KRT83, LRRC29, PLEKHA4, KRTAP1-5, LINC01552, MXRA7, IGFL2, ZNF319, RUNDC3A-AS1, RP11-800A3.4, MIR31HG, SMIM10L2A, KRT85, TMEM234, RP11-699A5.2, TRAK2, AC011718.2, IFNWP2, TG, KRTAP3-2, AF131215.9, COL4A3, TMEM265, ZMYM6NB, AC084809.2, RP4-569M23.2, PEF1, LA16c-380H5.6, C1orf115, ISCU, PBXIP1, VAMP8, KRT35, NMRK1, AC241585.2, GINM1, AP1M2, KRTAP1-1, MYL12B, MEGF9, SRL, KRTAP4-1, LPCAT2, FAM230B, MRPL40, CORO6, AC079630.4, KRTAP10-6, KRTAP5-3, ZNF208, PRRG2, AC079630.2, DVL1, LDOC1, RPS6KA2, RP11-638I2.6, CPQ, KRTAP26-1, CLTA, ABHD14A, KLHL2, ZFP36L1, CTD-2308L22.1, CTD-3157E16.2, ICAM5, UNC5C, PIP5KL1, CTSH, DMD, ANKS6, SGMS1, LINC01571, CTC-518B2.12, CLDN16, TMEM30B, MPG, CRLF1, HEG1, RP11-387M24.5, FSCN1P1, SLC22A31, SLC25A29, KIAA2013, FAM150A, PGBD3, ITSN2, AC008132.14, EFEMP2, AC008781.7, IGBP1, INPP5J, ATOH8, RP11-643A5.3, UBXN6, LA16c-380H5.5, CADM1, B3GALT4, RP11-351M8.2, TRAPPC3, FAM47E, RGAG4, CGB8, KRTAP2-2, TMEM43, NEBL-AS1, RAB11B, PTPN21, FAM49A, RP11-214C8.2, NAV2-AS1, BPY2C, ELMO3, PPL, PRKD2, NT5E, RBMS2, KRTAP5-4, HYAL2, LURAP1, NAV2, LBH, EMC10, ANXA3, TMEM163, CTC-281F24.3

Lung cancer: overlap CST6-related genes between MMDs lung cancer and TCGA lung cancer (LUAD and LUSC) datasets; Melanoma: overlap CST6-related genes between MMDs melanoma and TCGA SKCM datasets; Renal cancer: overlap CST6-related genes between MMDs renal cancer and TCGA renal cancer (KICH, KIRC and KIRP) datasets; Pan-cancer: CST6-related genes in TCGA pan-cancer level.

Table S5. Correlation results between expression of CST6 and epithelial cell infiltration, EMT and proliferation marker.

cancer	Corr (epi)	P (epi)	Corr (EMT)	P (EMT)	Corr (pro)	P (pro)
ACC	0.1883	1.01E-01	-0.1445	2.26E-01	-0.1805	1.16E-01
BLCA	0.2644	6.17E-08	-0.0350	4.89E-01	0.0290	5.59E-01
BRCA	0.2774	9.55E-21	0.1923	4.05E-10	-0.0982	1.15E-03
CESC	0.4914	7.00E-20	0.1218	3.88E-02	-0.0593	3.02E-01
CHOL	0.3643	2.90E-02	0.1735	3.34E-01	0.1413	4.11E-01
COAD	0.0790	1.81E-01	0.2374	6.19E-05	-0.1251	3.38E-02
DLBC	0.4380	2.07E-03	0.2949	5.49E-02	-0.1929	1.94E-01
ESCA	0.3727	2.37E-07	0.1246	1.22E-01	-0.2169	3.36E-03
GBM	0.4633	1.63E-09	0.1076	2.06E-01	-0.2432	2.45E-03
HNSC	0.4664	2.46E-29	-0.1247	5.13E-03	-0.2952	7.06E-12
KICH	0.4160	5.13E-04	0.0687	5.93E-01	0.1408	2.59E-01
KIRC	0.3967	2.01E-21	-0.0710	1.16E-01	0.0544	2.11E-01
KIRP	0.5793	3.27E-27	-0.1270	3.36E-02	-0.0460	4.36E-01
LGG	0.2875	3.82E-11	0.1730	1.03E-04	0.0931	3.58E-02
LIHC	0.4052	5.20E-16	-0.0069	8.97E-01	0.2173	2.55E-05
LUAD	0.2890	2.52E-11	0.0551	2.20E-01	0.0259	5.59E-01
LUSC	0.4541	1.04E-26	0.0239	5.99E-01	-0.2305	1.98E-07
MESO	0.6395	2.62E-11	-0.5245	8.28E-07	-0.2779	9.15E-03
OV	0.2074	1.88E-05	0.0379	4.47E-01	-0.0253	6.05E-01
PAAD	0.4134	9.74E-09	0.1709	3.35E-02	0.2018	6.90E-03
PCPG	0.1407	6.19E-02	0.0382	6.35E-01	-0.0497	5.11E-01
PRAD	0.3316	3.58E-14	0.2488	5.11E-08	0.0408	3.65E-01
READ	-0.1196	2.56E-01	0.3307	1.75E-03	-0.1476	1.60E-01
SARC	0.1587	1.07E-02	-0.1532	1.80E-02	-0.1210	5.22E-02
SKCM	0.8367	6.66E-28	-0.6875	3.87E-15	-0.0513	6.09E-01
STAD	0.1405	4.19E-03	0.1111	2.65E-02	-0.1706	4.88E-04
TGCT	0.1972	1.63E-02	-0.0507	5.46E-01	-0.0544	5.11E-01
THCA	0.6906	1.18E-72	0.3142	5.64E-12	0.1938	1.18E-05
THYM	0.3019	8.48E-04	0.1724	8.79E-02	-0.3477	1.07E-04
UCEC	0.3769	1.84E-07	-0.2250	2.76E-03	-0.2250	2.39E-03
UCS	0.5577	6.57E-06	-0.5148	8.03E-05	-0.2820	3.35E-02
UVM	0.2002	7.70E-02	-0.1223	2.93E-01	-0.3669	8.81E-04

Corr (epi): Spearman correlation coefficient between CST6 and epithelial cell; P (epi): p value of correlation analysis between CST6 and epithelial cell; Corr (EMT): Partial correlation coefficient between CST6 and EMT considering tumor purity as concomitant variable; P (EMT): p value of correlation analysis between CST6 and EMT; Corr (pro): Spearman correlation coefficient between CST6 and proliferation; P (epi): p value of correlation analysis between CST6 and proliferation.

Table S6. Spearman correlation results between EMT score and tumor purity.

Cancer	Correlation coefficient	P_value
ACC	-0.1657	1.55E-01
BLCA	-0.6106	7.82E-42
BRCA	-0.3971	1.00E-40
CESC	-0.3851	1.01E-11
CHOL	-0.4719	3.67E-03
COAD	-0.5658	2.91E-25
DLBC	0.3141	3.35E-02
ESCA	-0.3300	2.29E-05
GBM	-0.2879	4.90E-04
HNSC	-0.3295	2.97E-14
KICH	-0.2586	3.61E-02
KIRC	-0.4306	9.30E-24
KIRP	-0.4237	9.34E-14
LGG	0.1315	3.17E-03
LIHC	-0.5196	5.23E-26
LUAD	-0.5064	6.41E-34
LUSC	-0.4508	6.64E-26
MESO	-0.3582	1.02E-03
OV	-0.5295	8.75E-31
PAAD	-0.2397	2.42E-03
PCPG	-0.4849	8.20E-11
PRAD	-0.3119	4.64E-12
READ	-0.4091	6.25E-05
SARC	-0.3073	1.15E-06
SKCM	0.0785	4.33E-01
STAD	-0.4648	6.08E-23
TGCT	-0.1078	1.94E-01
THCA	-0.3509	7.78E-15
THYM	-0.2083	3.56E-02
UCEC	-0.3730	2.94E-07
UCS	0.2133	1.14E-01
UVM	-0.3042	6.42E-03

Table S7. Detailed information of CST6-related cis-eQTL from PancanQTL

cancer	SNP ID	SNP position	Alleles (A/a)	Beta	t-stat	P value
BRCA	rs7128076	65759634	T/C	-0.13	-3.55	0.000399
THCA	rs527737	65884800	C/T	0.12	3.36	0.000846
THCA	rs801738	65924217	C/G	0.13	3.37	0.00081
THCA	rs2576	65808467	C/T	0.12	3.37	0.000802
THCA	rs801742	65914766	C/A	0.13	3.39	0.000757
THCA	rs373488733	66018693	C/T	0.13	3.41	0.000713
THCA	rs801733	65934549	A/C	0.13	3.41	0.000707
THCA	rs484983	65880463	G/A	0.12	3.41	0.000699
THCA	rs144304307	65805825	C/CTGGCCA	0.12	3.42	0.000691
THCA	rs1151530	65862952	G/A	0.12	3.51	0.000494
THCA	rs537497	65866187	G/A	0.12	3.51	0.000494
THCA	rs571374	65817592	C/T	0.12	3.51	0.000489
THCA	rs2241303	65819661	C/A	0.13	3.58	0.000379
THCA	rs576740	65852571	G/A	0.13	3.67	0.000269
THCA	rs493320	65832367	C/A	0.13	3.76	0.000194
THCA	rs539046	65843778	A/T	0.13	3.76	0.000191
STAD	rs11545221	65746136	G/T	0.42	3.8	0.000166
STAD	rs12575004	65748116	C/T	0.42	3.8	0.000166
STAD	rs72928843	65743510	G/A	0.42	3.8	0.000166
STAD	rs72928860	65747740	C/T	0.42	3.8	0.000166
STAD	rs72928879	65752327	T/G	0.42	3.8	0.000166
STAD	rs72928884	65754124	C/T	0.42	3.8	0.000166
STAD	rs72928898	65757290	C/T	0.42	3.8	0.000166
STAD	rs72928900	65757387	C/T	0.42	3.8	0.000166
STAD	rs57968007	65729680	T/C	0.4	3.82	0.000155
STAD	rs58905199	65729677	T/C	0.4	3.82	0.000155
STAD	rs115607690	65763296	A/T	0.42	3.82	0.000154
STAD	rs12574568	65762654	T/C	0.42	3.82	0.000154
STAD	rs2276017	65766092	C/T	0.42	3.82	0.000154
STAD	rs72930914	65760584	A/G	0.42	3.82	0.000154
STAD	rs72930933	65763919	T/C	0.42	3.82	0.000154
STAD	rs72930942	65765074	G/A	0.42	3.82	0.000154
STAD	rs72930951	65766360	G/A	0.42	3.82	0.000154
STAD	rs72930952	65766671	A/G	0.42	3.82	0.000154
STAD	rs80186982	65766266	T/G	0.42	3.82	0.000154
STAD	rs60109456	65742283	A/G	0.18	3.84	0.000146
STAD	rs72930945	65765552	G/A	0.43	3.85	0.000139
STAD	rs7128076	65759634	T/C	0.19	3.87	0.000129
THCA	rs2430978	65816656	G/C	0.14	3.87	0.000126
STAD	rs117619207	65768814	G/T	0.42	3.88	0.00012
STAD	rs144765366	65768761	G/A	0.42	3.88	0.00012
STAD	rs2277305	65769159	G/C	0.42	3.88	0.00012

STAD	rs376636768	65770312	TAGAG/T	0.42	3.88	0.00012
STAD	rs3825068	65768093	A/G	0.42	3.88	0.00012
STAD	rs72930964	65770403	T/C	0.42	3.88	0.00012
STAD	rs72930967	65770953	G/A	0.42	3.88	0.00012
STAD	rs72930974	65771913	G/T	0.42	3.88	0.00012
THCA	rs576836	65823569	G/C	0.14	3.88	0.000117
STAD	rs148479829	65753833	C/T	0.43	3.92	0.000106
THCA	rs1642958	65821973	G/C	0.14	4.03	6.58E-05
STAD	rs12576095	65776305	G/A	0.44	4.05	6.23E-05
STAD	rs12577165	65776429	T/C	0.44	4.05	6.23E-05
THCA	rs577013	65832973	T/C	0.15	4.16	3.82E-05
THCA	rs574335	65833266	C/T	-0.15	-4.27	2.31E-05
READ	rs543952	65744275	T/C	-0.66	-4.56	2.19E-05
READ	rs622779	65744247	G/A	0.66	4.56	2.19E-05
THCA	rs570760	65833631	C/T	0.16	4.51	8.01E-06
THCA	rs559161	65761637	A/G	-0.19	-4.63	4.68E-06
COAD	rs117619207	65768814	G/T	0.77	4.75	3.32E-06
COAD	rs144765366	65768761	G/A	0.77	4.75	3.32E-06
COAD	rs2277305	65769159	G/C	0.77	4.75	3.32E-06
COAD	rs376636768	65770312	TAGAG/T	0.77	4.75	3.32E-06
COAD	rs3825068	65768093	A/G	0.77	4.75	3.32E-06
COAD	rs72930964	65770403	T/C	0.77	4.75	3.32E-06
COAD	rs72930967	65770953	G/A	0.77	4.75	3.32E-06
COAD	rs72930974	65771913	G/T	0.77	4.75	3.32E-06
THCA	rs1786171	65769809	G/C	-0.2	-4.77	2.40E-06
THCA	rs55644043	65772310	TAATA/T	-0.2	-4.8	2.15E-06
THCA	rs677350	65761967	G/A	-0.22	-4.93	1.12E-06
THCA	rs9736731	65760813	C/T	-0.22	-4.93	1.12E-06
THCA	rs1786172	65770658	A/G	-0.22	-4.95	1.04E-06
THCA	rs502363	65775592	C/T	-0.22	-5.01	7.75E-07
THCA	rs610497	65765551	C/T	-0.21	-5.02	7.15E-07
THCA	rs534201	65776728	A/T	-0.22	-5.03	6.85E-07
THCA	rs543952	65744275	T/C	-0.23	-5.04	6.53E-07
THCA	rs507672	65767153	C/T	0.22	5.05	6.28E-07
THCA	rs1151510	65756167	T/C	-0.23	-5.07	5.82E-07
THCA	rs1151511	65756283	A/G	-0.23	-5.07	5.82E-07
THCA	rs1191716	65754458	T/C	-0.23	-5.07	5.82E-07
THCA	rs522553	65771669	T/C	-0.23	-5.07	5.65E-07
THCA	rs549334	65774659	C/G	-0.23	-5.07	5.65E-07
THCA	rs1151514	65758854	G/A	-0.23	-5.07	5.63E-07
THCA	rs1204011	65741086	G/A	0.23	5.12	4.43E-07
THCA	rs506873	65767215	A/C	0.23	5.14	4.07E-07
THCA	rs586921	65747701	G/A	0.23	5.15	3.81E-07
THCA	rs622779	65744247	G/A	0.24	5.24	2.39E-07

THCA	rs645571	65752852	A/T	0.25	5.28	1.98E-07
THCA	rs540584	65765866	C/G	0.24	5.34	1.43E-07
THCA	rs1192184	65773382	A/G	0.24	5.35	1.36E-07
THCA	rs590531	65773939	C/A	0.24	5.35	1.36E-07
THCA	rs619701	65772080	G/A	0.24	5.35	1.36E-07

CST6 position (hg19): chr11, 65779312-65780976, +; CST6-related cis-eQTLs were defined if the SNP was within 1 Mb from the CST6 transcriptional start site (TSS); eQTL analysis was performed by Matrix eQTL in linear regression model.
